# OpiCa1 Modulates Cardiomyocyte Viability Through PI3K/Akt Inhibition with Minimal Systemic Impact Beyond RyR Targeting

**DOI:** 10.3390/toxins17110550

**Published:** 2025-11-04

**Authors:** Xiaofen Ma, Xiaoyu Hua, Xiao Peng, Zhixiao Yang, Yi Wang, Qian Zhang, Lianbo Wang, Shumin Wang, Héctor H. Valdivia, Liang Xiao, Mei Wang

**Affiliations:** 1 Xinjiang Key Laboratory of Natural Medicines Active Components and Drug Release Technology, Engineering Research Center of Xinjiang and Central Asian Medicine Resources, Ministry of Education, School of Pharmacy, Xinjiang Medical University, Ürümqi 830000, China; 2 Faculty of Naval Medicine, Naval Medical University, Shanghai 200433, China; 3 Key Laboratory of Biological Defense Ministry of Education, Second Military Medical University, Shanghai 200433, China; 4 Shanghai Key Laboratory of Medical Bioprotection, Second Military Medical University, Shanghai 200433, China; 5 College of Traditional Chinese Medicine, Yunnan University of Chinese Medicine, Kunming 650500, China; 6 College of Animal Science and Veterinary Medicine, Shanxi Agricultural University, Jinzhong 030801, China; 7 Department of Medicine and Cardiovascular Research Center, University of Wisconsin-Madison School of Medicine and Public Health, Madison, WI 53726, USA

**Keywords:** OpiCa1, ryanodine receptor, Ca^2+^, apoptosis, transcriptomics, PI3K/Akt signaling

## Abstract

Calcins represent a class of novel peptide ligands for ryanodine receptors (RyRs), demonstrating therapeutic potential against Ca^2+^ dysregulation-related cardiac diseases. Nevertheless, their biological effects beyond RyR modulation and underlying mechanisms remain unexplored. This study employed Opicalcin1 (OpiCa1), the most bioactive calcin member, revealing that while it reduced cytosolic Ca^2+^ in H9c2 cardiomyocytes, it concurrently diminished cell viability and promoted apoptosis. Transcriptomics and Western blot analyses identified suppression of the negatively regulatory PI3K/Akt pathway as the mechanistic basis. In acute/chronic in vivo studies, high-dose OpiCa1 (≥50 mg/kg i.v.) exhibited minimal impact on body weight, histopathology, and organ indices, while accompanied with subtle alterations in serum indicators, including slight elevations in AST, ALT, and LDH, alongside mild reductions in CK-MB and TBIL-Z. These findings unveil OpiCa1’s modulation on cardiomyocyte viability through PI3K/Akt inhibition with minimal systemic impact, providing new insights into non-RyR-mediated actions of calcins and critical toxicological support for developing calcin-based therapies targeting Ca^2+^-dysregulated cardiac pathologies.

## 1. Introduction

Scorpions, one of the most ancient and widely distributed animals in the world, have a long history of medicinal use. The dried body of the scorpion, known as Quanxie in traditional Chinese medicine, is a valuable and esteemed crude drug with significant therapeutic applications [1,2,3]. Calcins, a class of scorpion venom-derived peptide toxins (3.7–4.2 kDa) [4], possess a distinctive inhibitory cystine knot (ICK) motif stabilized by three disulfide bonds. Their unique architecture features asymmetric clustering of basic residues, forming a bipolar globular structure with pronounced cationic polarity [5,6,7]. This structural signature confers two critical functions: (1) cell membrane penetration capability, and (2) specific binding and partial activation of ryanodine receptors (RyRs). By modulating RyR-mediated calcium release from the sarcoplasmic reticulum, calcins influence cardiac ECC (excitation-contraction coupling) [8,9,10]. This regulatory mechanism underlies their therapeutic potential for counteracting both primary (e.g., catecholaminergic polymorphic ventricular tachycardia) [11,12,13] and secondary calcium dysregulation-related arrhythmias [14,15,16].

While current research primarily focuses on calcins as high-specificity RyR ligands leveraging electrostatic and structural complementarity between their cationic arginine/lysine clusters and the RyR pore region, their pronounced amphipathic nature suggests broader interaction potential beyond RyRs [8]. The asymmetric distribution of positive charges on these globular molecules theoretically enables non-specific binding to diverse biomacromolecules (e.g., proteins, nucleic acids, polysaccharides, lipids) [17,18,19] containing anionic domains or pore structures, potentially mediating biological effects extending beyond calcium homeostasis and ECC [20,21,22]. Although RyRs remain the only confirmed high-affinity targets with defined pharmacological activity, identifying additional molecular targets and their impacts on non-ECC cellular networks represents a critical research frontier [23,24,25]. Addressing this knowledge gap is essential for comprehensively evaluating calcins’ polypharmacological profiles and their therapeutic potential as novel cardiovascular agents.

Building upon these structural and pharmacological foundations, we systematically investigated OpiCa1, the most bioactive calcin variant, through a tripartite experimental paradigm: (i) in vitro cardiomyocyte assays assessing viability, apoptosis, and calcium dynamics; (ii) in vivo dose–response profiling via tail vein administration; and (iii) integrated transcriptomic analysis of cardiac tissue and isolated cardiomyocytes. Our findings reveal that OpiCa1 exerts low-toxicity cardiomyocyte suppression potentially mediated by PI3K/Akt pathway modulation, a mechanism distinct from canonical RyR-mediated ECC. This discovery not only expands the understanding of calcin’s polypharmacological effects beyond RyRs and calcium homeostasis, but also provides critical toxicological evidence for developing RyR-targeting antiarrhythmic agents.

## 2. Results

### 2.1. OpiCa1-Induced Modulations in Cardiomyocytes

**Figure 1:**
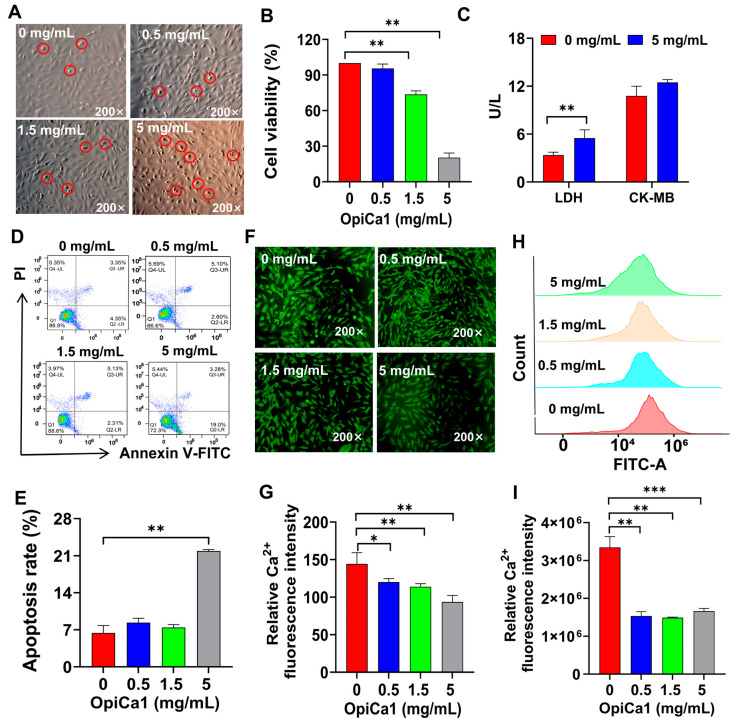
**Functional Evaluation of OpiCa1 in H9c2 Cardiomyocytes.** (**A**) Morphological map of cells intervening with 0–5 mg/mL OpiCa1. Red circles indicate morphologically abnormal cells. (**B**) Cell viability of Opica1 on H9c2 cells. *n* = 6–8. (**C**) LDH and CK-MB released by OpiCa1. *n* = 6. (**D**,**E**) H9c2 cell apoptosis tested by flow cytometry. *n* = 3. (**F**,**G**) Fluorescence images of intracellular Ca^2+^ detected by Fluo-4 AM. *n* = 3. (**H**,**I**) Intracellular Ca^2+^ tested by flow cytometry with Fluo-4 AM. *n* = 3. Data are presented as mean ± SEM. Statistical analysis was performed using Student’s *t*-test. * *p* < 0.05; ** *p* < 0.01; *** *p* < 0.001.

At OpiCa1 concentrations ranging from 0 to 5 mg/mL, the number of shrunken H9c2 cardiomyocytes showed a significant positive dose-dependent correlation (Figure 1A). In contrast, cell viability exhibited a marked negative correlation, with the viability of H9c2 cells at 5 mg/mL being less than 30% of that in the control group (Figure 1B). Regarding cardiomyocyte damage indicators, both LDH and CK-MB showed a mild increase, among which LDH displayed statistical significance (Figure 1C). Flow cytometry analysis revealed an apoptosis rate of 20% at an OpiCa1 concentration of 5 mg/mL (Figure 1D,E). Meanwhile, as a peptide ligand for RyR, we also examined and found that OpiCa1 reduced Ca^2+^ concentration by both fluorescence microscopy (Figure 1F,G) and flow cytometry (Figure 1H,I). These results suggest that OpiCa1 inhibits cardiomyocyte viability and promote cell apoptosis beyond influencing cytosolic Ca^2+^.

### 2.2. OpiCa1 Inhibits the PI3K/Akt Signaling Pathway in Cardiomyocytes

**Figure 2:**
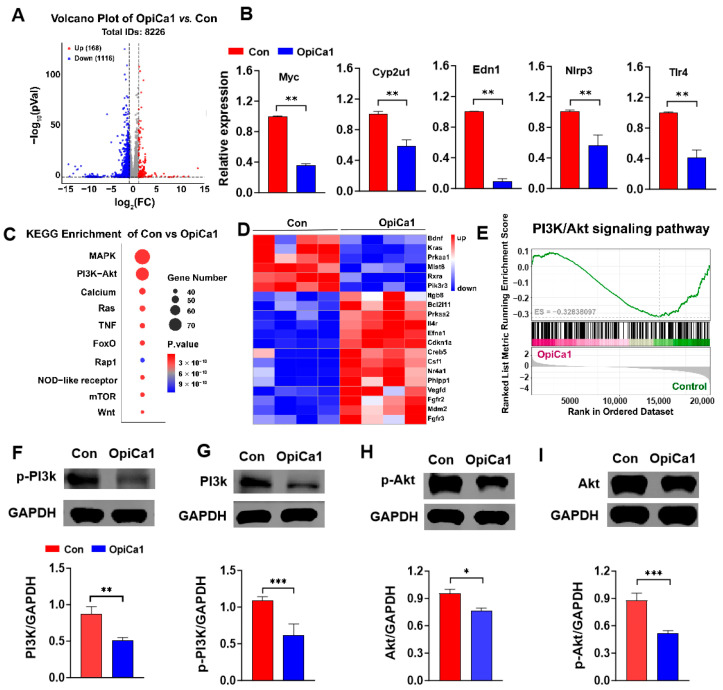
**Transcriptomic analysis of H9c2 cells intervened with OpiCa1.** (**A**) Volcano diagram of differential genes in H9c2 cells after OpiCa1 treatment. Vertical and horizontal dashed lines denote |Log_2_FC| > 0.378 and *p* value < 0.05. (**B**) Five randomly selected genes were verified by RT-qPCR. *n* = 6–8. (**C**) KEGG pathway analysis of the H9c2 cell transcriptomes affected by OpiCa1. (**D**) Heatmap of differentially expressed genes in H9c2 cells. (**E**) GSEA plot of PI3K/Akt signaling pathway. ES: Enrichment Score. ES > 0: positive correlation. ES < 0: negative correlation. (**F**–**I**) Western blotting of PI3K/p-PI3K and Akt/p-Akt. *n* = 3–6. Data are presented as mean ± SEM. Statistical analysis was performed using Student’s *t*-test. * *p* < 0.05; ** *p* < 0.01; *** *p* < 0.001.

Transcriptomic sequencing showed that 8226 genes were detected in H9c2 cardiomyocytes after OpiCa1 intervention, among which only 168 genes were significantly upregulated while 1116 genes were obviously downregulated (Figure 2A). Five randomly selected downregulated genes were verified by RT-qPCR, and the results were consistent with those of the transcriptomic analysis (Figure 2B). KEGG pathway enrichment analysis demonstrated that the target genes were predominantly associated with the mitogen-activated protein kinase (MAPK) pathway and phosphatidylinositol 3-kinase (PI3K)/protein kinase B (Akt) pathway, and were additionally implicated in the calcium signaling pathway (Figure 2C). Further heatmap analysis of signaling pathway-related the 20 differentially expressed genes in OpiCa1-treated cells compared to controls, with 6 downregulated and 14 upregulated (Figure 2D). Consistent with the results of OpiCa1 inhibiting the viability of H9c2 cardiomyocytes and promoting apoptosis, the PI3K/Akt signaling pathway, which exerts a negative regulatory effect, was inhibited by OpiCa1, with an ES value of −0.328 (Figure 2E). Western blot analysis revealed that OpiCa1 significantly inhibited the protein expression and phosphorylation levels of PI3K/p-PI3K (Figure 2F,G) and Akt/p-Akt (Figure 2H,I). These results suggest that OpiCa1 inhibit the PI3K/Akt signaling pathway, which may contribute to the inhibitory effect of OpiCa1 on cardiomyocytes.

### 2.3. OpiCa1 Exhibits Low In Vivo Biological Toxicity

**Figure 3:**
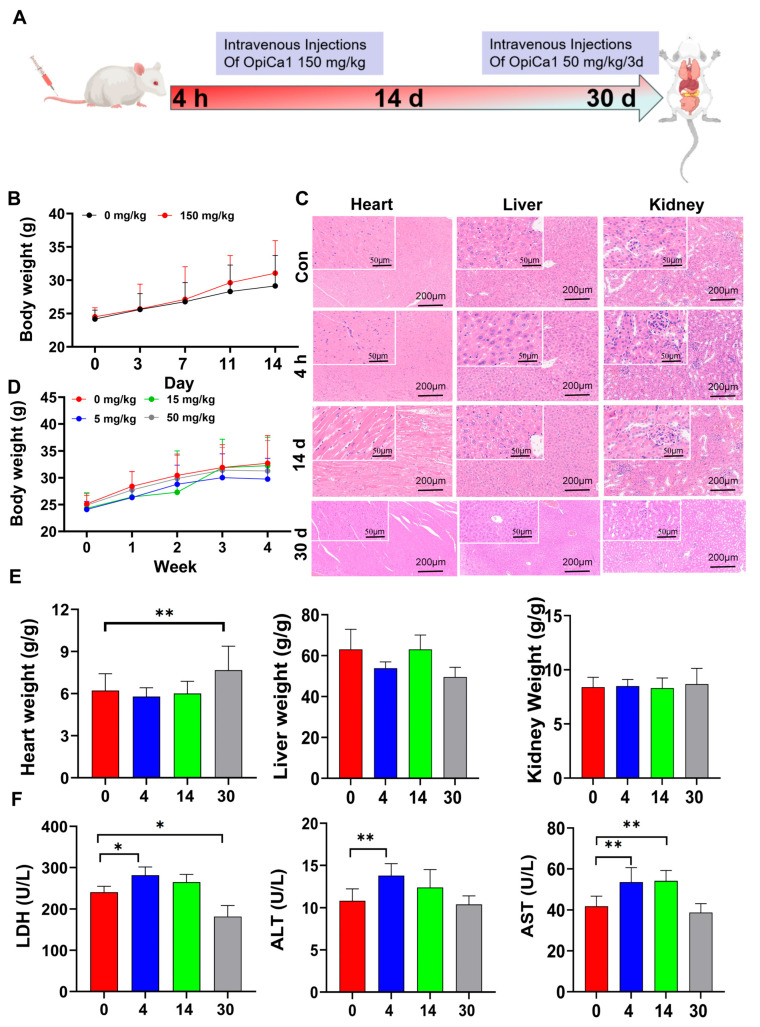
**In vivo biological effects of OpiCa1 in intact mice.** (**A**) Schematic diagram of OpiCa1’s biological effects in vivo after administration. (**B**) Statistics of body weight changes in mice of 14 d. *n* = 10–12. (**C**) H&E staining of heart, liver and kidney. *n* = 3. (**D**) Statistics of body weight changes in mice of 30 d. *n* = 10–12. (**E**) Statistics of organ coefficient. *n* = 10–12. (**F**) Blood biochemical indices. *n* = 5. Data are presented as mean ± SEM. Statistical analysis was performed using ANOVA. * *p* < 0.05; ** *p* < 0.01.

To further investigate the in vivo biological effects of OpiCa1, we administered OpiCa1 at different concentrations and frequencies via tail vein injection (0~150 mg/kg), and monitored the body weight, organ indices, as well as pathological and blood biochemical indicators of mice over 4 h, 14 days, and 30 days (Figure 3A). The results revealed that there was no significant change in the body weight of mice within 14 days after a single injection of a super-high dose of OpiCa1 (150 mg/kg). Similarly, within the dose range of 0~50 mg/kg, administered every 3 days, there was also no significant change in the body weight of mice over 30 days (Figure 3B,D). Other general condition indicators, such as diet, drinking water, and urination, showed no notable changes (Supplementary Table S1). Whether administered a single super-high dose of 150 mg/kg or administered at a dose of 50 mg/kg every 3 days, there were no pathological changes observed in major organs such as the heart, liver, and kidneys (Figure 3C). Except for a slight increase in the cardiac index in the group administered 50 mg/kg OpiCa1 every 3 days, there were no significant changes in organ indices across all doses (Figure 3E). Blood biochemical tests revealed that after a single super-high dose of 150 mg/kg, LDH, ALT, and AST levels were slightly elevated at 4 h. After 14 days, except for a slight elevation in AST, LDH and ALT levels partially recovered, showing no significant difference compared to the control group (Supplementary Figure S1). In contrast, administration of 50 mg/kg OpiCa1 every 3 days only exhibited a slight decrease in LDH levels (Figure 3F). Furthermore, all other biochemical indicators of organs showed no significant changes. These results suggest that OpiCa1, at a super-high dose (150 mg/kg i.v.), may cause slight changes in blood biochemical indicators. However, long-term administration of high doses of OpiCa1 appears to have negligible toxic effects on the body.

### 2.4. OpiCa1 Inhibits the Cardiac PI3K/Akt Signaling Pathway in Intact Mice

**Figure 4:**
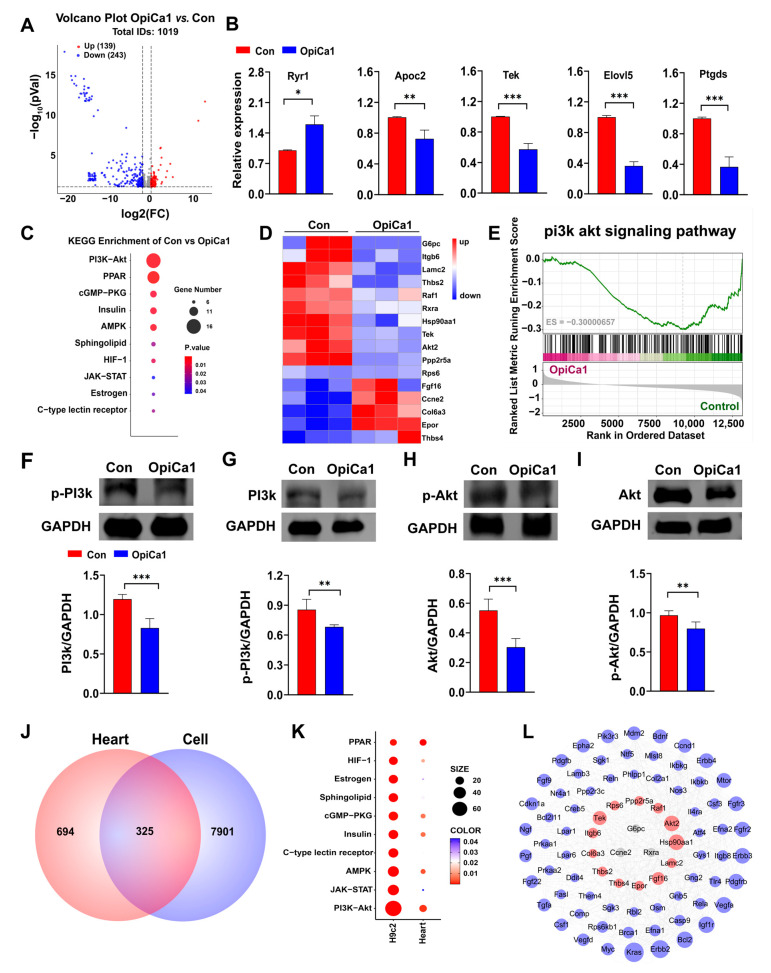
**Transcriptomic analysis of hearts intervened with OpiCa1.** (**A**) Volcano diagram of differential genes in hearts after OpiCa1 treatment. Vertical and horizontal dashed lines denote |Log_2_FC| > 0.378 and *p* value < 0.05. (**B**) Five randomly selected genes were verified by RT-qPCR. *n* = 6–8. (**C**) Heatmap of differentially expressed genes in hearts. (**D**) KEGG pathway analysis of the hearts transcriptomes affected by OpiCa1. (**E**) GSEA plot of PI3K/Akt signaling pathway. (**F**–**I**) Western blotting of PI3K and Akt. *n* = 3–6. (**J**) Venn diagram of differentially expressed genes in H9c2 cells and hearts. (**K**) Pathway enrichment analysis of KEGG differentially significant genes in H9c2 cells and the hearts. (**L**) Protein–protein interaction network of differentially expressed genes in PI3K-Akt signaling pathways on H9c2 cells and hearts. (Blue markers: differential genes derived from heart, Red markers: differential genes derived from H9c2 cells, Gray markers: differential genes shared by both. The larger the circle, the higher the node degree). Data are presented as mean ± SEM. Statistical analysis was performed using Student’s *t*-test. * *p* < 0.05; ** *p* < 0.01; *** *p* < 0.001.

We administered a dose of 50 mg/kg OpiCa1 every 3 days, and after 30 days, the hearts were dissected for transcriptome sequencing. The results showed that among the 1019 genes identified, 139 genes were upregulated and 243 genes were downregulated (Figure 4A). Similarly, five genes were randomly selected for RT-qPCR, and gene expression results consistent with the transcriptome were obtained (Figure 4B). KEGG pathway enrichment analysis showed that the PI3K/Akt signaling pathway was the top enriched pathway (Figure 4C). Heatmap analysis revealed that among the top 16 differentially expressed genes, 11 genes were downregulated and 5 genes were upregulated (Figure 4D). Meanwhile, GSEA analysis also indicated that the PI3K/Akt signaling pathway was inhibited, with an ES value of −0.300 (Figure 4E). Consistent with cardiomyocytes, the expression levels and phosphorylation levels of PI3K and Akt proteins in heart tissue were significantly decreased (Figure 4F,I). A joint analysis of the transcriptomes affected by OpiCa1 on H9c2 cardiomyocytes and mouse hearts revealed 325 common differentially expressed genes between the two, and the PI3K/Akt signaling pathway was the top enriched pathway (Figure 4J). Through protein–protein interaction analysis, it was found that genes G6pc, Ccne2, and Rxra in the PI3K/Akt signaling pathway played a central role in the effects of OpiCa1 on cardiomyocytes and heart tissue (Figure 4K,L). In summary, OpiCa1 also exerted a significant inhibitory effect on the PI3K/Akt signaling pathway in intact mice.

## 3. Discussion

Studies indicate calcins’ selective RyR binding and partial activation are mediated by their ICK-fold stabilized bipolar structure with clustered cationic residues [13,25,26], enabling modulation of cardiomyocyte Ca^2+^ homeostasis and ECC for potential cardiac therapy. Nevertheless, investigations into non-RyR targets, driven by toxicological profiling during novel antiarrhythmic development, motivate exploration of calcins’ extra-RyR biological effects and mechanisms.

This study shows that OpiCa1 lowers cytosolic Ca^2+^ concentration by partially activating RyR2, which elicits limited Ca^2+^ release from the SR. The elevated cytosolic Ca^2+^ is subsequently extruded via plasma membrane Na^+^-Ca^2+^ exchangers while SR Ca^2+^-ATPase mediates Ca^2+^ reuptake into SR stores [27,28]. This coordinated action of Na^+^/Ca^2+^ exchanger(NCX) -mediated extrusion and Sarco/Endoplasmic Reticulum Ca^2+^ ATPase(SERCA)-dependent reuptake collectively contributes to the observed decrease in cytosolic Ca^2+^ levels [29,30,31]. Notably, OpiCa1 exhibited dose-dependent effects within 0–5 mg/mL range by reducing cardiomyocyte viability, increasing LDH release, and significantly elevating apoptotic cell population at 5 mg/mL. These results suggest calcin exerts cardiomyocyte inhibitory effects, though it remains unclear whether this inhibition stems primarily from Ca^2+^ reduction or involves additional intracellular signaling pathways.

This study shows that transcriptomic analysis was utilized to characterize the impacts of OpiCa1 on cardiomyocyte gene expression, revealing that downregulated genes are more prevalent than upregulated ones, a finding consistent with its inhibitory properties. Notably, both MAPK and PI3K/Akt signaling pathways exhibited inhibitory correlations, with MAPK typically promoting pro-inflammatory and pro-apoptotic responses [32,33], while PI3K/Akt exerts protective inhibitory functions. Consequently, OpiCa1’s suppression of the PI3K/Akt pathway aligns mechanistically with its attenuation of cardiomyocyte viability and induction of apoptosis. Experimental validation confirmed inhibition of key PI3K and Akt protein expression and phosphorylation, further substantiating calcin’s inhibitory effect on this pathway. Collectively, these results suggest that calcin-induced cardiomyocyte suppression may operate through PI3K/Akt pathway attenuation rather than solely via Ca^2+^ modulation.

Notwithstanding these findings, our in vivo investigations into the acute and chronic toxicological and biological effects of OpiCa1 revealed a remarkably benign safety profile. Administration of a high intravenous dose (≥50 mg/kg) every 3 days for 30 days elicited minimal alterations in body weight or histopathological parameters across major organs, with only the heart exhibiting mild hypertrophy. Serum biochemistry analyses indicated transient perturbations: minor elevations in ALT, AST, and LDH levels contrasted with subtle downward trends in CK-MB and Total Bilirubin (TBIL-Z). Collectively, these marginal biochemical deviations underscore calcin’s capacity to exert significant biological activity in vivo while eliciting minimal systemic toxicity [34]. This favorable safety profile is likely attributable to calcin’s inherent physicochemical properties as a compact, globular polypeptide, facilitating rapid target engagement followed by swift metabolic clearance and elimination [35,36,37]. Consequently, calcins represent promising low-toxicity leads for both RyR-targeted arrhythmia therapies and non-canonical applications exploiting PI3K/Akt pathway modulation.

As an intracellular calcium release channel, the functional activity of RyR_2_ is dependent on calcium signal homeostasis. The PI3K pathway can indirectly regulate the phosphorylation state and opening efficiency of RyR_2_ by modulating downstream molecules such as Calcium/Calmodulin-Dependent Protein Kinase II (CaMKII). Since this study is the first to focus on the core role of the PI3K/Akt pathway in mediating the effects of calcin, experimental validation of other mechanisms was not performed herein. In future work, we plan to systematically explore the interactions between RyR_2_ and multiple signaling pathways through integrated multi-omics analysis and investigations at the molecular and protein levels. This will collectively clarify the comprehensive regulatory effects of calcin on cellular functions and lay a more robust foundation for prospective research on calcin.

## 4. Conclusions

Within the dosage range of this study, comprehensive evaluations at cellular and animal levels indicate that OpiCa1 exhibits low or no toxicity towards cardiomyocytes and the heart. Consequently, this research concludes that the OpiCa1 peptide is a lead candidate peptide with high safety and low hepatic and renal toxicity, categorized as a practical min-toxic compound. It exerts certain effects on intracellular calcium ion concentrations and cardiac function indicators. This study will provide some scientific basis for the future development of new drugs for calcin peptides in cardiovascular diseases and help to further develop their clinical value.

## 5. Materials and Methods

### 5.1. OpiCa1 Synthesis

OpiCa1 was commercially obtained from Shanghai Qiangyao Biological Technology Co., Ltd., (Shanghai, China). The peptide was initially synthesized as linear calcin via Fmoc (9-fluorenylmethoxycarbonyl) solid-phase peptide synthesis (SPPS27.0) methodology. Subsequent oxidative folding under native conditions in folding buffer facilitated the formation of intramolecular disulfide bridges, resulting in cyclization of the peptide structure. Following gradient purification by reversed-phase high-performance liquid chromatography (RP-HPLC), the final product demonstrated a high purity of 95.36% as Validated by analytical HPLC (Agilent, california, USA).

### 5.2. Cell Viability Assay

The H9c2 (STCC30008, Wuhan Zishan Biological Co., Ltd., Wuhan, China, 10–20 generations) cardiomyocyte cell line was maintained in Dulbecco’s Modified Eagle Medium (DMEM) supplemented with low sodium bicarbonate, 100 U/mL penicillin-streptomycin, and 10% fetal bovine serum (FBS) under standard culture conditions (37 °C, 5% CO_2_). For experiments, cells were seeded in 96-well plates at a density of 5 × 10^3^ cells per well and allowed to adhere for 12 h. After the attachment period, cells were treated with 100 μL of OpiCa1 at varying concentrations (0.5, 1.5, and 5 mg/mL) for 4 h, while serum-free DMEM served as the negative control. Following treatment, the medium was replaced with fresh DMEM containing 10% CCK-8 reagent (Tao Shu, Shanghai, China, C0005), and cells were incubated for an additional 1 h at 37 °C. All experiments were performed with three biological replicates and six technical replicates. Absorbance was measured at 450 nm using a microplate reader (Biotek, VT, USA), and cell viability was calculated as follows:

$$\mathrm{Cell}\;\mathrm{Viability}\left(\%\right)=\frac{\left({\mathrm{OD}}_{450,\mathrm{treated}}-{\mathrm{OD}}_{450,\mathrm{blank}}\right)}{\left({\mathrm{OD}}_{450,\mathrm{control}}-{\mathrm{OD}}_{450,\mathrm{blank}}\right)}\times 100$$

### 5.3. LDH and CK-MB Determination

H9c2 cardiomyocytes were seeded in 6-well plates at a density of 5 × 10^4^ cells per well and cultured for 12 h to ensure proper adherence. Subsequently, cells were treated with 2 mL of OpiCa1 at graded concentrations (0.5, 1.5, and 5 mg/mL) for 4 h, while an equal volume of serum-free DMEM served as the vehicle control. Following incubation, the culture supernatant was collected and centrifuged (1000× *g*, 10 min) to remove cellular debris. LDH and CK-MB levels were quantified using commercial assay kits (Jiancheng Bio, Nanjing, China) according to the manufacturer’s protocols. Briefly, the working reagent was prepared and mixed with the supernatant, followed by spectrophotometric analysis using a fully automated biochemistry analyzer (BK-280, Shangdong, China). All experiments were performed with three biological replicates and six technical replicates.

### 5.4. Flow Cytometry

H9c2 cardiomyocytes were plated in 6-well culture plates at a density of 5 × 10^4^ cells per well and allowed to adhere for 12 h. Cells were then treated with 2 mL of OpiCa1 at concentrations of 0.5, 1.5, and 5 mg/mL for 4 h, with serum-free DMEM serving as vehicle control. Following treatment, cell pellets were collected. For calcium flux analysis, cell pellets were resuspended in 500 μL of Fluo-4 AM (Beyotime Bio, Shanghai, China, S1061S) working solution prepared according to the manufacturer’s protocol. The cell suspension was incubated at 37 °C for 30 min under light-protected conditions to allow complete dye loading. First, exclude debris using FSC-A/SSC-A, select single cells using FSC-H/FSC-A, then using a dual-staining protocol with FITC-Annexin V and propidium iodide (PI). Compensation controls were established by staining untreated cells with either FITC-Annexin V or PI alone. Experimental and control groups were stained with FITC-Annexin V/PI cocktail and incubated for 30 min at 37 °C in the dark. Fluorescence intensity (for calcium measurement) and apoptosis rates were quantified using a CytoFLEX LX flow cytometer (Beckman Coulter, Suzhou, China). Data acquisition was performed with CytExpert software2.4, and subsequent analysis was conducted using FlowJo software10.8.

### 5.5. Fluorescence Microscopy Imaging

H9c2 cardiomyocytes were seeded in 6-well plates at a density of 5 × 10^4^ cells per well and cultured for 12 h in complete DMEM medium supplemented with 10% fetal bovine serum. Cells were then treated with 2 mL of OpiCa1 at graded concentrations (0.5, 1.5, and 5 mg/mL) for 4 h, with serum-free DMEM serving as the vehicle control. Following treatment, cells were harvested by gentle trypsinization and centrifugation. The cell pellet was resuspended in 500 μL of Fluo-4 AM working solution and incubated at 37 °C for 30 min under light-protected conditions to allow complete dye loading. After incubation, cells were washed twice with PBS to remove excess dye and immediately imaged using an inverted fluorescence microscope (ZEISS Axio Observer Vert.1, Oberkochen, Germany) equipped with a FITC filter set (excitation 488 nm/emission 516 nm). Fluorescence intensity reflects intracellular calcium ion concentration with fluorescence values quantified using ImageJ1.8.0. All experiments included 3 biological replicates (*n* = 3).

### 5.6. Animals and Treatment

Equal numbers of male and female ICR mice (6–8 weeks old, weighing 18–22 g, 60 mice, China) were purchased from Shanghai Jiesijie Experimental Animal Co., Ltd. (Shanghai, China) Mice were housed in ventilated cages at 22–23 °C with 55–60% relative humidity and a 12-h light/dark cycle. Water and standard chow pellets were available ad libitum. All animal care and experimental procedures strictly followed the guidelines of the ARRIVE and were approved by the Institutional Animal Ethics Committee of the Naval Medical University. All mice were randomly assigned to groups using a random number table, comprising a control group and a drug-treated group, with 10–12 animals in each group.

Based on the effective dose identified in previous studies [20], mice were administered 150 mg/kg of OpiCa1 via tail vein injection. After 14 days of observation, the mice were anesthetized, and blood samples as well as heart, liver, and kidney tissues were collected to evaluate the acute effects of OpiCa1. For the assessment of chronic effects, OpiCa1 was administered via tail vein injection at doses of 5, 15, and 50 mg/kg, respectively, with an interval of 3 days between injections. At 4 h, 14 days, and 30 days after the initiation of treatment, the mice were anesthetized, followed by the collection of blood, heart, liver, and kidney tissues for subsequent analyses. In each group, an equal volume of PBS was injected as a control. The body weight of the mice was recorded every 3 days throughout the experiment. During the observation period, the mice had free access to food and water and were housed under standard environmental conditions.

### 5.7. Blood Biochemical Analysis

After anesthesia, blood samples from mice in each group subjected to acute and chronic treatments were collected via orbital sinus. The blood samples were allowed to stand at room temperature for 2 h, then centrifuged at 3000 rpm for 10 min, and the supernatants were harvested. Wuhan Servicebio Technology Co., Ltd. (Wuhan, China) was commissioned to detect the changes in serum indicators including ALT, AST, TBIL-Z, LDH, CK, CK-MB, Cr, BUN, DBIL-Z, and Ca^2+^ using the single-reagent method with an automatic biochemistry analyzer (Bokang Bio-industry Co., Ltd., Shangdong, China, BK-280), with *n* = 5. Serum biochemical indicators including ALT, AST, and LDH were measured using the fully automated biochemical analyser. To ensure data accuracy, the instrument was calibrated using original manufacturer’s calibration standards prior to each batch of sample testing. Additionally, two levels of commercial quality control materials were incorporated throughout the testing process for monitoring. Only when the quality control results fell within the predefined range was the data from that batch accepted.

### 5.8. Histological Analyses

After anesthetizing the mice, heart, liver, and kidney tissues were collected from each group of mice subjected to acute and chronic treatments. These tissues were fixed in 4% paraformaldehyde solution, embedded in paraffin, and sectioned into slices with a thickness of 5 μm. The sections were stained with hematoxylin and eosin (H&E), and the histopathological changes in tissues of each group were observed using a Nikon Eclipse E600 microscope (Nikon Corporation, Tokyo, Japan).

### 5.9. RNA Sequencing and Analysis

H9c2 cells treated with the indicated drugs were collected from 6-well plates. An appropriate amount of tissue (approximately 50–100 mg) was harvested. The total RNA extraction procedure strictly followed the manufacturer’s kit operating guidelines. RNA sequencing was carried out by LC-Bio Technologies Hangzhou Co., Ltd. (Hangzhou, China), and analyzing transcriptome data using Lianchuan Bio’s cloud platform. All experiments were performed with four biological replicates.

### 5.10. Bioinformatics Analysis

All transcriptome genes were fully aligned using unigenes, the NCBI database (http://www.ncbi.nlm.nih.gov/, accessed on 24 September 2025), the GO database (http://www.geneontology.org, accessed on 24 September 2025), the SwissProt database (http://www.expasy.ch/sprot/, accessed on 24 September 2025), and the KEGG database (http://www.genome.jp/kegg/, accessed on 24 September 2025). Differentially expressed genes were identified and subjected to GO analysis. Functional gene pathways and expression profiles were analyzed by comparison with the KEGG database. ES: Enrichment score, whose core function is to quantify the degree to which the expression of a specific gene set deviates from random distribution across two or more sample groups, thereby determining whether that gene set is associated with the studied phenotype.

### 5.11. RT-qPCR Validation

H9c2 cells were seeded in 6-well plates at a density of 5 × 10^4^ cells per well and cultured for 12 h. Then, 2 mL of 5 mg/mL OpiCa1 was added for incubation for 4 h, with an equal volume of serum-free DMEM medium used as the control. After incubation, cell pellets were collected, and total RNA from each group of cells was extracted using an RNA extraction kit (Fastagen, Shanghai, China, Cat. 220010). For myocardial tissue, 20–50 mg of tissue was weighed, homogenized using a tissue homogenizer, and the supernatant was collected. Total RNA was then extracted via the Trizol method.

After determining the RNA concentration, reverse transcription was performed using Reverse TraAce^®^ qPCR RT Master Mix (TOYOBO, Tokyo, Japan, KMM-101). Quantitative PCR was conducted using SYBER^®^ Green Realtime PCR Master Mix and gene-specific primers on the qPCR system of Eppendorf Master Cycler realplex 2 (Eppendorf, Hamburg, Germany). The gene expression levels of each group were calculated and compared according to the 2^−∆∆Ct^ method. All specific primers were designed and synthesized by Sangon Biotech Co., Ltd. (Shanghai, China), as detailed in Table 1.

### 5.12. Western Blot

Protein extraction and Western blotting were performed according to the method described in the literature [38]. Briefly, cardiomyocytes from each group and mouse hearts were collected, and total proteins from cells and tissues were extracted using a mixture consisting of SDS cell lysis buffer (Beyotime, Shanghai, China, P0013G), 1× protease inhibitor, phosphatase inhibitor, and PMSF (Solarbio, Beijing, China, P0100,). Protein samples were quantified using a standard BCA assay kit (Beyotime, Shanghai, China, P0010). Protein separation was performed by 12% SDS-PAGE (12%) at 200 V for 30 min. Proteins were transferred onto NC membranes at 200 mA for 80 min at 4 °C, and then the NC membranes were blocked with protein-free rapid blocking solution at room temperature for 1 h. The NC membranes were incubated with specific primary antibodies overnight at 4 °C; the detailed primary antibodies are as follows: PI3K/p-PI3K, Akt/p-Akt (HuaAn Bio, Hangzhou, China, ET1608-70, ET1607-73, HA721672, ET1609-51), and GAPDH (abclone, Wuhan, China, A19056). On the next day, the membranes were washed 3 times with TBST and incubated with secondary antibody (HRP-conjugated goat anti-rabbit IgG, ABclonal) at room temperature for 1 h. Hypersensitive ECL luminescent solution (Meilun Bio, Liaoning, China, MA0186) was used to detect the target protein bands, which were subsequently quantified using Image J.

### 5.13. Statistical Analysis

Data are expressed as mean ± SEM. Statistical analyses were performed using SPSS12.0 software, including one-way analysis of variance (one-way ANOVA) for comparisons among multiple groups and independent-samples *t*-test for comparisons between two groups. The significance level was set at α = 0.05, and a *p* < 0.05 was considered statistically significant. Flow cytometry plots were generated using FlowJo software10.8, and statistical graphs were plotted using GraphPad Prism software9.0. All instances of ‘*n*’ labeled in the figures denote the number of biological replicates, referring to independent cell cultures from different batches or distinct animal individuals.

## Figures and Tables

**Table 1:** Primer Sequence List.

	Forward Primer (5′ → 3′)	Reverse Primer (5′ → 3′)	Species
GAPDH	AGTCCACTGGCGTCTTCACC	TGATCTTGAGGCTGTTGTCATACTTC	Rat
Camk1g	GAACGGTACACCTGCGAGAAAG	GCTTGCCTCCACTTGCTCTTG	Rat
Slc8a3	CCGCATGGTGGATATGAGTGTTC	CTGCTATTCTCTTGGCTTCCTCTTC	Rat
Sphk1	GTACGAGCAGGTGACTAATGAAGAC	AGGACAGACTGAGCACAGAATAGAG	Rat
Edn1	TTCTGCCACCTGGACATCATCTG	AACGCTTGGACCTGGAAGAACC	Rat
Tlr4	CCGCTTTCACCTCTGCCTTCAC	ACCACAATAACCTTCCGGCTCTTG	Rat
Nlrp3	GCCGTCTACGTCTTCTTCCTTTCC	CATCCGCAGCCAGTGAACAGAG	Rat
Pim1	GCTGCTCAAGGACACAGTCTACAC	CGTGGTAGCGATGGTAGCGAATC	Rat
Kcnj8	ACAAGCACGGACCTCCTACATTG	ACACGCCCTCCTCCTCAGTC	Rat
Cyp2u1	CAGCAGCTTCGACGAGGACTAC	CAGCAGGCACCAGAGCAGAG	Rat
β-actin	CTGCCGCATCCTCTTCCTC	TGCCACAGGATTCCATACCC	Mouse
RyR1	ATCGTCATTCTGCTGGCTATCATTC	CCTTCACTTGCTCTTGTTGGTCTC	Mouse
Apoc2	TTCCTGGCTCTATTCCTGGTCATC	TGGCAACCTCCTTGGCAGAG	Mouse
Ptgds	TCAATCTCACCTCTACCTTCCTCAG	AGTGGATGCTGCCCGAGTG	Mouse
Tek	GTGCTGTTGGCGTTTCTGATTATG	TGGTTCTTCTCTGTTCTGGAATGC	Mouse
Elovl5	CGTCCTCTGGTGGTACTACTTCTC	CGGTGATCTGGTGGTTGTTCTTG	Mouse

## Data Availability

The original contributions presented in this study are included in the article/Supplementary Material. Further inquiries can be directed to the corresponding authors.

## Supplementary Materials

The following supporting information can be downloaded at: https://www.mdpi.com/article/10.3390/toxins17110550/s1, Figure S1: Blood biochemistry tests and RT-qPCR verified genes; Table S1: General observations after single and repeated administration in mice.

## Abbreviations

AST	Aspartate Aminotransferase
ALT	Alanine Aminotransferase
LDH	Lactate Dehydrogenase
BCA	Bicinchoninic Acid Assay
CK-MB	Creatine Kinase—Myocardial Band
CCK-8	Cell Counting Kit-8
CaMKII	Calcium/Calmodulin-Dependent Protein Kinase II
DBIL-Z	Direct Bilirubin—Z
DEPC	Diethyl pyrocarbonate
ECC	excitation-contraction coupling
ECL	Enhanced ChemiLuminescence
FITC	Fluorescein isothiocyanate
GAPDH	Glyceraldehyde-3-phosphate dehydrogenase
GSEA	Gene Set Enrichment Analysis
H&E	Hematoxylin and Eosin
KEGG	Kyoto Encyclopedia of Genes and Genomes
MAPK	Mitogen-Activated Protein Kinase
NC	Nitrocellulose membrane
OpiCa1	Opicalcin1
PMSF	Phenylmethylsulfonyl fluoride
RyR_2_	Ryanodine Receptor 2
PI	Propidium iodide
RyRs	Ryanodine receptors
ROS	Reactive Oxygen Species
SDS	Sodium Dodecyl Sulfate
SR	Sarcoplasmic reticulum
SERCA	Sarco/ Endoplasmic Reticulum Ca^2+^ ATPase
TBST	Tris-Buffered Saline with Tween 20
TBIL-Z	Total Bilirubin-Z
