# Supplementary material for: OpiCa1 Modulates Cardiomyocyte Viability Through PI3K/Akt Inhibition with Minimal Systemic Impact Beyond RyR Targeting
Source: Toxins (Basel). 2025 Nov 4;17(11):550. doi: 10.3390/toxins17110550 (PMC12656661; doi:10.3390/toxins17110550)
Supplement: Supplementary file 1 [file toxins-17-00550-s001.zip › toxins-3923516-supplementary.pdf]

Supplementary Materials

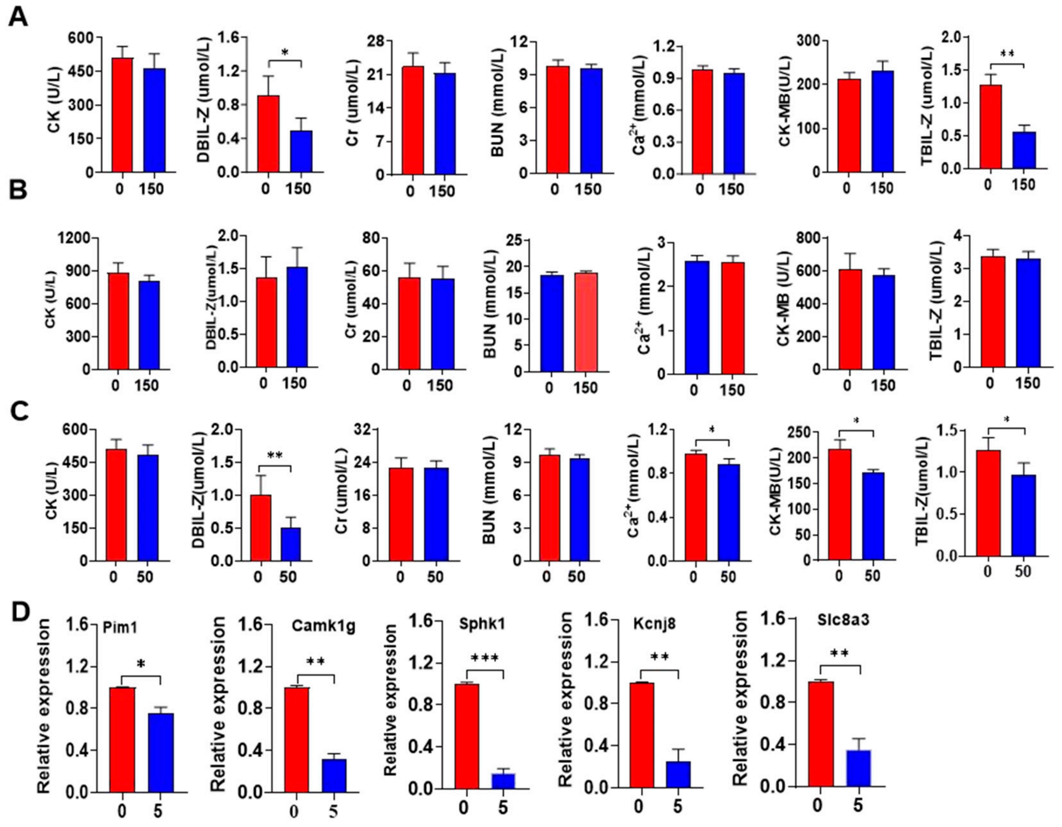

Figure S1. Blood biochemistry tests and RT-qPCR verified genes. (A) Comparison of blood biochemical indices in OpiCa1 and Con group mice at 4 h days. n=6-8. **(B)** Comparison of blood biochemical indices in OpiCa1 and Con group mice at 14 d days.n=6-8. **(C)** Comparison of blood biochemical indices in OpiCa1 and Con group mice at 30 d days.n=6-8. (D) RT-qPCR verified genes. n = 6-8. Data are presented as means ±S. Statistical analysis was performed using Student's t-test. *p < 0.05; **p < 0.01;***p< 0.001**.**

Table S1: General observations after single and repeated administration in mice.

No.	General Observation	General Indications
1	Nostrils, breathing, body surface	Normal respiratory rate and depth, no cyanosis on the body surface
2	Athletic ability	Decrease in spontaneous activity within 30 min; animals were lethargic but could be awakened by pinprick or external disturbance to resume voluntary activity, and returned to normal activity after 30 min
3	Convulsions, reflexes	No convulsions, reflexes or other reactions
4	Eyelid indications	Normal pupils and eyeballs without tearing or inflammation
5	Cardiovascular indications	Electrocardiogram shows normal heart rate and no characteristic systole or diastole of blood vessels
6	Hair erection, salivation, nociception, dystonia	No such phenomenon
7	Faeces, vomit, urine, skin	Stool colour and hardness are normal, urine colour and frequency are normal, no skin redness, swelling, allergy, etc.
